# Let-7c inhibits cholangiocarcinoma growth but promotes tumor cell invasion and growth at extrahepatic sites

**DOI:** 10.1038/s41419-018-0286-6

**Published:** 2018-02-14

**Authors:** Yu Xie, Hang Zhang, Xing-Jun Guo, Ye-Chen Feng, Rui-Zhi He, Xu Li, Shuo Yu, Yan Zhao, Ming Shen, Feng Zhu, Xin Wang, Min Wang, Asha Balakrishnan, Michael Ott, Feng Peng, Ren-Yi Qin

**Affiliations:** 1 Department of Biliary-Pancreatic Surgery, Affiliated Tongji Hospital, Tongji Medical College, Huazhong University of Science and Technology, 1095 Jiefang Avenue, Wuhan, Hubei 430030 China; 2 Department of Gastroenterology, Hepatology, and Endocrinology, Hannover Medical School (MHH), TWINCORE, Center for Experimental and Clinical Infection Research, Feodor-Lynen-Straße 7, 30625 Hannover, Germany

## Abstract

Cholangiocarcinoma (CCA) is a cancer type with high postoperative relapse rates and poor long-term survival largely due to tumor invasion, distant metastasis, and multidrug resistance. Deregulated microRNAs (miRNAs) are implicated in several cancer types including CCA. The specific roles of the miRNA let-7c in cholangiocarcinoma are not known and need to be further elucidated. In our translational study we show that microRNA let-7c expression was significantly downregulated in human cholangiocarcinoma tissues when compared to adjacent tissues of the same patient. Let-7c inhibited the tumorigenic properties of cholangiocarcinoma cells including their self-renewal capacity and sphere formation in vitro and subcutaneous cancer cell growth in vivo. Ectopic let-7c overexpression suppressed migration and invasion capacities of cholangiocarcinoma cell lines in vitro, however, promoted distant invasiveness in vivo. Furthermore, we found that let-7c regulated the aforementioned malignant biological properties, at least in part, through regulation of EZH2 protein expression and through the DVL3/β-catenin axis. The miRNA let-7c thus plays an important dual role in regulating tumorigenic and metastatic abilities of human cholangiocarcinoma through mechanisms involving EZH2 protein and the DVL3/β-catenin axis.

## Introduction

Cholangiocarcinoma (CCA) is acknowledged as being difficult to diagnose and treat. Advanced stage of the disease at diagnosis, early extensive invasion and distant metastasis, as well as the multi-drug resistance of the local tumor^1^ contribute to poor survival rates^2^. The overall 5-year survival rate is <5%^3^. The progression of cholangiocarcinoma involves multiple genetic and epigenetic alterations^4^. In order to find novel and effective therapies, it is necessary to explore the underlying molecular mechanisms of the disease^5^.

MiRNAs function as post-translational regulators of protein coding mRNA expression leading to inhibition of translation or mRNA degradation^6^. A single miRNA can interact with multiple target genes and thereby essentially regulates multiple cellular pathways. Several miRNAs were shown to be deregulated in cancers and to exert oncogenic or tumor-suppressive functions^7^. The members of let-7 family are highly conserved in sequence and function from *Caenorrhabditis elegans* to humans^8,9^ and are critical regulators of embryonic development, stem cell maintenance, differentiation, glucose metabolism, and the development of pathological processes including tumorigenesis^10^. Moreover, previous studies have suggested that members of the let-7 family function as tumor suppressors in various cancers including non-small cell lung cancer^11^, breast cancer^12^, hepatocellular carcinoma^13,14^, and pancreatic cancer^15,16^. However, only a few studies in cholangiocarcinoma were reported.

We have previously carried out miRNA profiling in cholangiocarcinoma tissues^17^ and found significant deregulation of let-7c. Let-7c was shown earlier to play a critical role in regulating migration and invasion of tumor cells^18^. Our current studies demonstrate that let-7c participates in regulating tumorigenesis of cholangiocarcinoma including tumor-initiating capacity and sphere formation. We also found that let-7c inhibits migration and invasion of cholangiocarcinoma cells, in vitro, by directly targeting the EZH2 protein. In addition, we reveal that let-7c enhances invasion and tumor growth of cholangiocarcinoma at distant sites in nude mice via the DVL3/β-catenin axis. The results thus elucidate partially antagonistic molecular mechanisms of let-7c in regulating cholangiocarcinoma.

## Results

### Expression of let-7c is differentially regulated in both tumor tissues and sera of cholangiocarcinoma patients

**Fig. 1:**
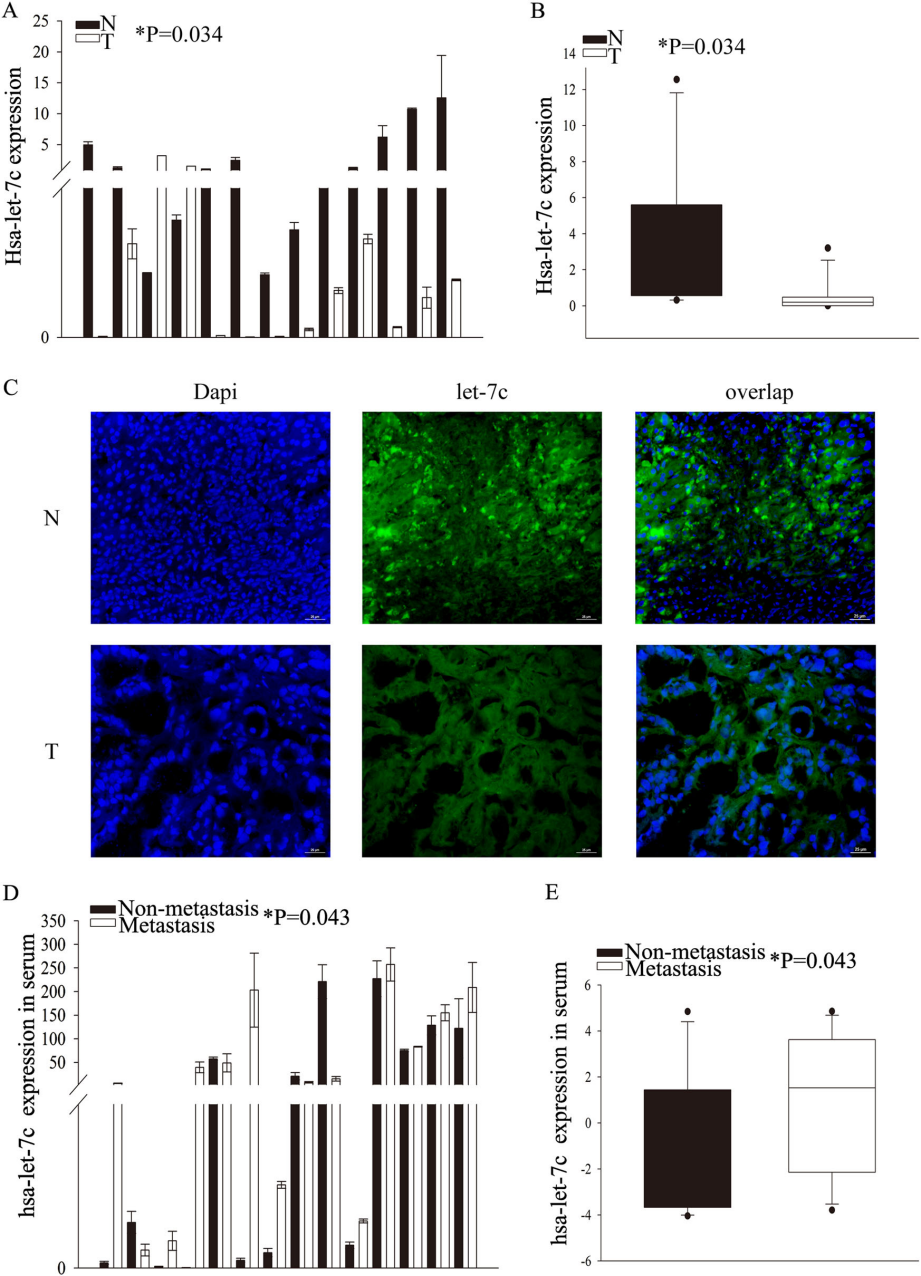
Expression of let-7c is differentially regulated in both tumor and sera of cholangiocarcinoma patients. **a** Expression of let-7c in 13 cholangiocarcinoma and paratumor tissues by RT-qPCR. **b** Collected data show the expression of let-7c in 13 cholangiocarcinoma and paratumor tissues. **c** Immunochemistry to evaluate the expression of let-7c in cholangiocarcinoma and paratumor tissues. **d** The level of let-7c in serum from distant metastatic patients and non-metastatic patients by RT-qPCR. **e** Collected data show the expression of let-7c in serum from distant metastatic patients and non-metastatic patients. *P < 0.05; *N* Normal bile duct tissue; T cholangiocarcinoma (tumor group)

In our preliminary study, we used Agilent miRNA microarrays to identify differentially expressed miRNAs in three pairs of human cholangiocarcinoma and paratumor tissues. We found 21 differentially expressed miRNAs. Let-7c was the most consistently and significantly deregulated^17^ and thus further verified in 13 cholangiocarcinoma and matched paratumor tissues, where let-7c showed lower levels in the cancer tissue (Fig. 1a, b). Furthermore, we performed in situ hybridization (ISH) to detect expression of let-7c in cholangiocarcinoma and matched paratumor tissues. These results showed that let-7c is expressed lower in cholangiocarcinoma than in matched paratumor tissues (Fig. 1c). Interestingly, in serum samples from the same patients, let-7c levels were higher in patients with metastatic disease than in patients without metastasis (Fig. 1d, e). We therefore selected let-7c for further study.

### Regulating the expression of let-7c can affect self-renewal of cholangiocarcinoma cells in vitro and tumorigenic potential in vivo

**Fig. 2:**
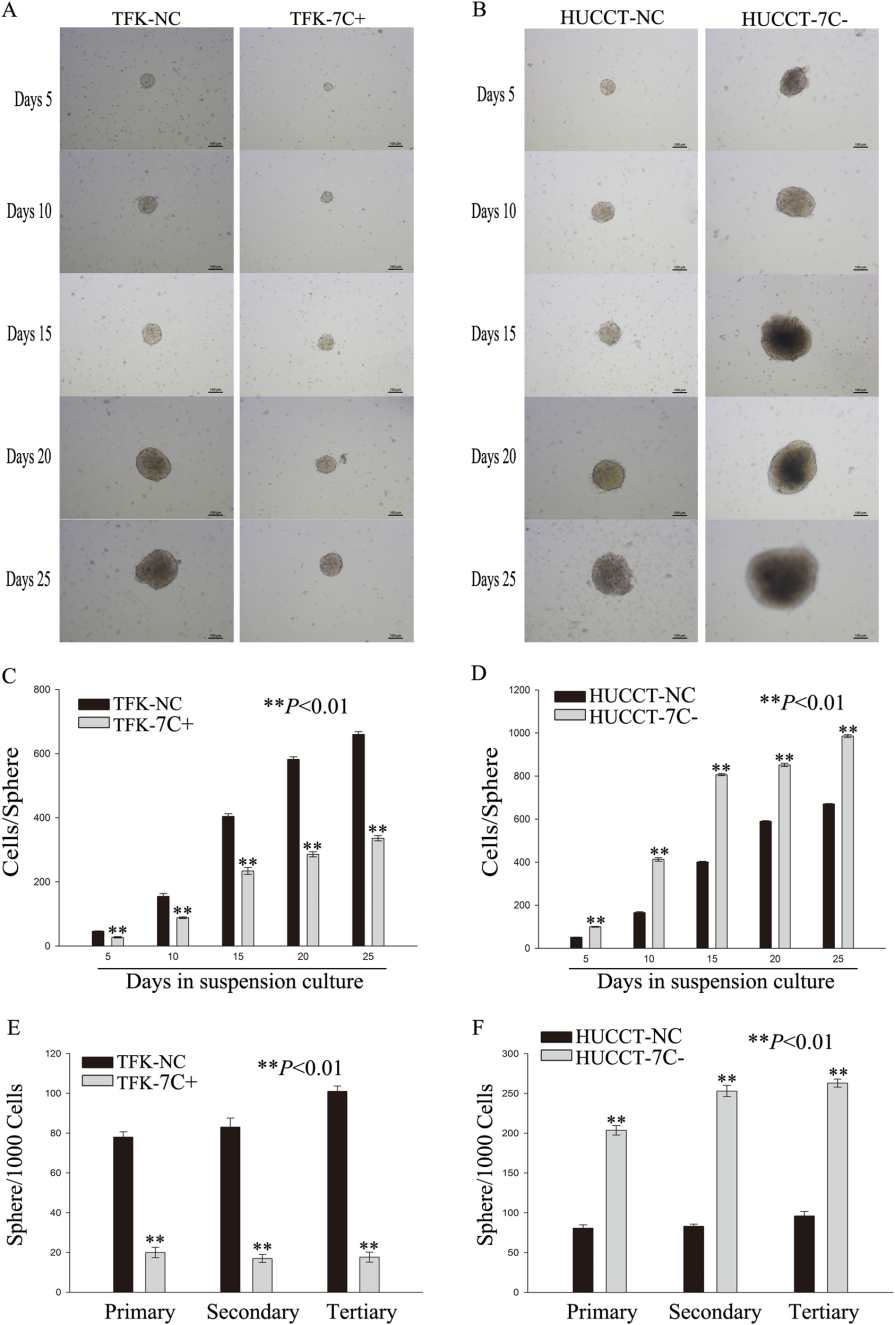
Regulating the expression of let-7c can affect self-renewal of cholangiocarcinoma cells in vitro. **a** Sphere formation with upregulated let-7c transduced TFK-1 cells at multiple time points. **b** Shpere formation with downregulated let-7c transduced HUCCT-1 cells at multiple time points.** c**,** d** Quantification of cell numbers per sphere. (**e** and **f**) The number of spheres per 1000 cells indicates the capacity for sphere formation. The data were compared with the negative control group. *P < 0.05, **P < 0.01. NC negative control group; 7C+ let-7c-upregulated group; 7c− let-7c-downregulated group

To determine whether ectopic expression of let-7c can influence cholangiocarcinoma tumorigenicity both in vitro and in vivo, we generated a stable TFK-1 cell line with a recombinant lentivirus overexpressing let-7c and a HUCCT-1 cell line with lentivirus-mediated inhibition of let-7c. In order to determine the role of let-7c in affecting the tumorigenic properties of cholangiocarcinoma cells, the above mentioned TFK-1 and HUCCT-1 cells were used to generate spheres in serum-free conditions. The self-renewing capacity of cells is indicated by the number of spheres in vitro, whereas the number of cells in every sphere reflects the self-renewal capacity of each clone, making up the sphere^12^. Twenty-five days later, the cells in the group with upregulated let-7c expression formed smaller spheres than the NC group (Fig. 2a, c), whereas the cells with downregulated let-7c expression formed larger spheres than the NC group (Fig. 2b, d). Over three passages, the overexpressed let-7c cells formed fewer spheres than the cells in the NC group (Fig. 2e), whereas the cells with downregulated let-7c formed more spheres than NC group (Fig. 2f). We plated the detached spheres on collagen IV in serum containing medium to simulate differentiation of cancer cells. After 6 days in these conditions, the expression levels of let-7c decreased after sphere formation and increased during differentiation (Supplementary Figure 4A). Taken together, the spheres formed by overexpressed let-7c cells showed slower growth rates and smaller sizes. Additionally, they could not be passaged as spheres. These results demonstrate that let-7c-overexpressing spheres may have undergone the first step toward losing their self-renewal capacity. In contrast, the spheres with low let-7c expression showed a greater ability for sphere formation, thereby revealing a higher self-renewal capacity.

**Fig. 3:**
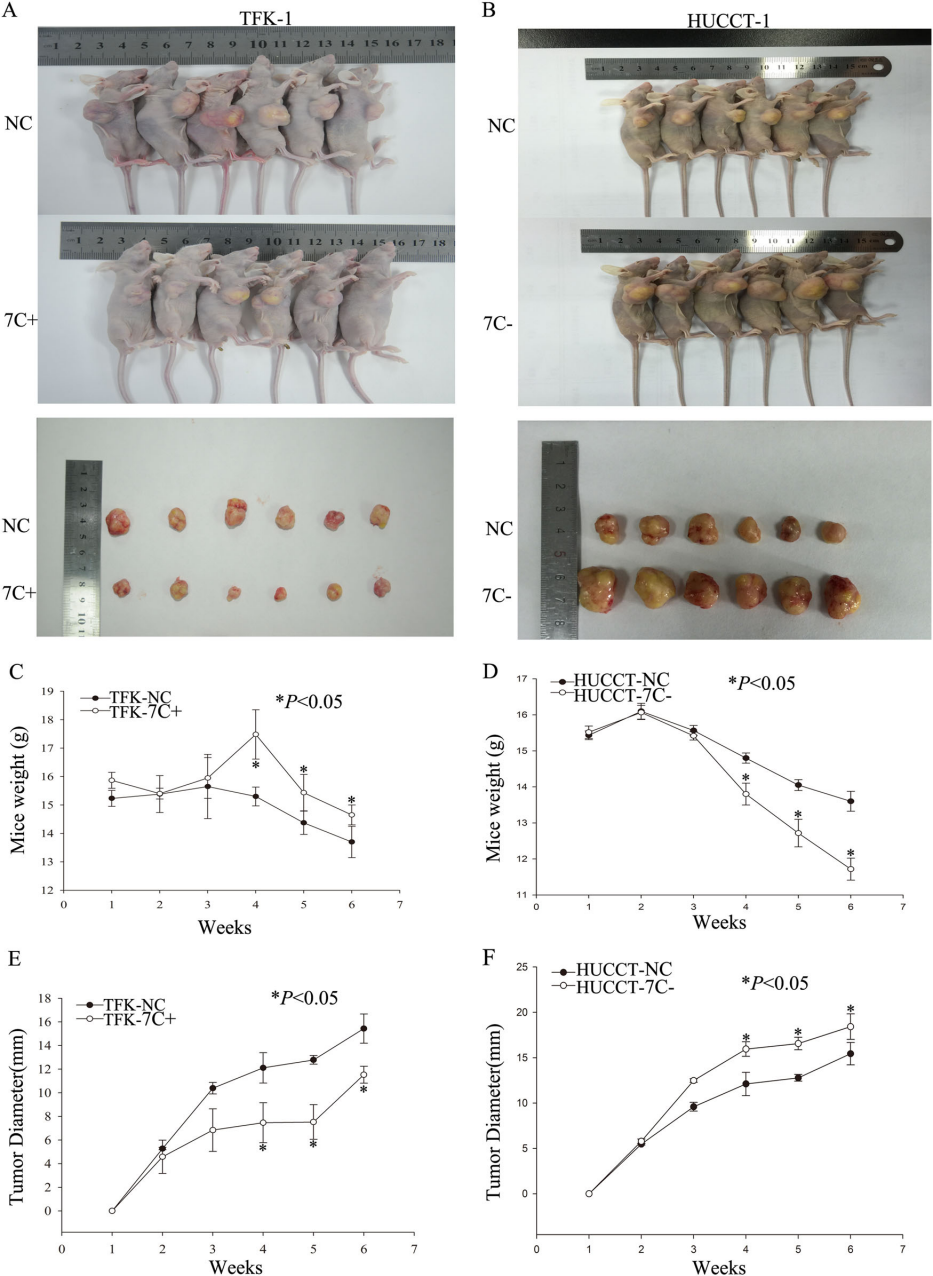
Regulating the expression of let-7c can affect tumorigenic potential in vivo. Tumorigenic capacities of transfected TFK-1 cells in BALB/c-nude mice, 6 weeks after subcutaneous injection. **b** Tumorigenic capacities of transfected HUCCT-1 cells in BALB/c-nude mice, 6 weeks after subcutaneous injection. **c**, **d** BALB /c nude mice weights. **e**, **f** BALB /c nude mice tumor diameter. *P < 0.05; NC negative control group; 7C+ let-7c-upregulated group; 7c− let-7c-downregulated group

We next injected the stable TFK-1 cell line (with increased let-7c expression and respective parental control cells) and HUCCT-1 cell line (with decreased let-7c expression and respective parental control cells) hypodermically into the right armpit of BALB/c nude mice. Three weeks later, the mice that were injected with let-7c upregulated cells showed smaller tumors at the injection site (Fig. 3a) compared to controls, while those injected cells with downregulated let-7c showed larger tumors compared with the control group (Fig. 3b). There were significant differences in weight of mice, from the fourth week between the two groups (Fig. 3c, d). Further, the cells, which overexpressed let-7c, formed tumors at a much slower rate than the control group. The cells with downregulated let-7c showed the opposite trend (Fig. 3e, f). The results thus indicate differential tumor-initiating abilities of cholangiocarcinoma cells following let-7c regulation.

### Aberrant expression of let-7c inhibits migration and invasion of cholangiocarcinoma cells in vitro but enhances metastasis in vivo

**Fig. 4:**
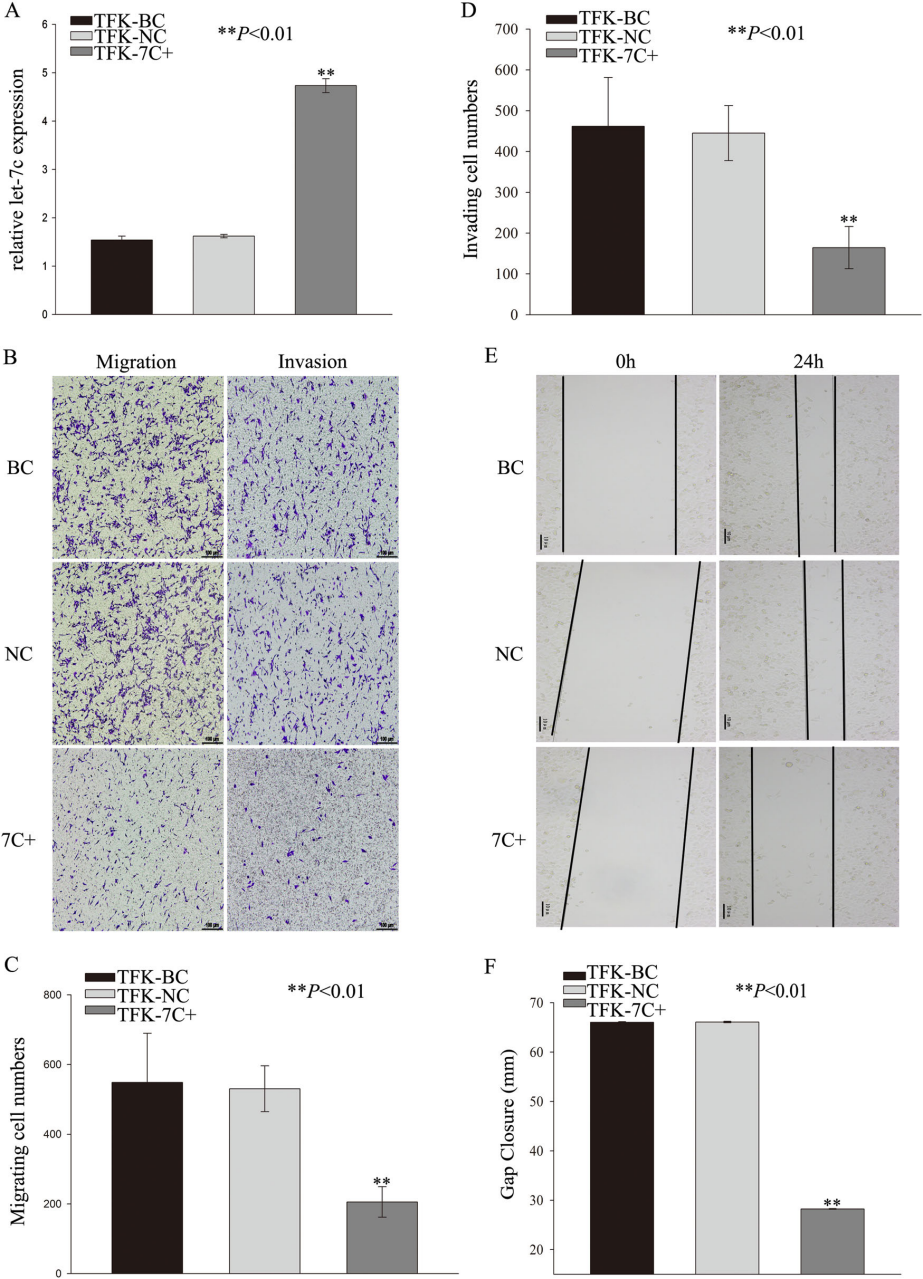
Aberrant expression of let-7c inhibits migration and invasion of cholangiocarcinoma cells in vitro. **a** Validation of let-7c expression in TFK-1 cells following treatment with mimic. **b** overexpressed let-7c inhibited invasive capacity of TFK-1 cells. Decreased migration and invasion in TFK-1 cells with upregulated let-7c expression. Numbers of (**c**) migrating and (**d**) invading cells. **e** Wound-healing assay with transfected TFK-1 cells. **f** The extent of gap closure in wound-healing assay showing. **P < 0.01. BC blank control group; NC negative control group; 7C+ let-7c-upregulated group

We next examined whether let-7c can affect the migration and invasion capacities of cholangiocarcinoma cells. We transfected TFK-1 cells with let-7c mimics and transfected HUCCT-1 cells with an inhibitor to increase or decrease let-7c expression, respectively. Let-7c levels of cell lines were analyzed by qRT-PCR and compared with scramble-transfected cells (Fig. 4a and Supplementary Figure 1A). Invasion assays showed that the overexpression of let-7c was associated with lower invasion rates compared to scramble control cells (Fig. 4b–d). TFK-1 cells, which were transfected with let-7c mimic, also showed decreased wound-healing ability when compared with the controls (Fig. 4e, f). In contrast, downregulation of let-7c expression in HUCCT-1 cells facilitated the invasion of cholangiocarcinoma cells (Supplementary Figure 1B, 1C and 1D) as well as significant gap closure in the wound-healing assay in comparison with the scramble control group (Supplementary Figure 1E and 1F).

**Fig. 5:**
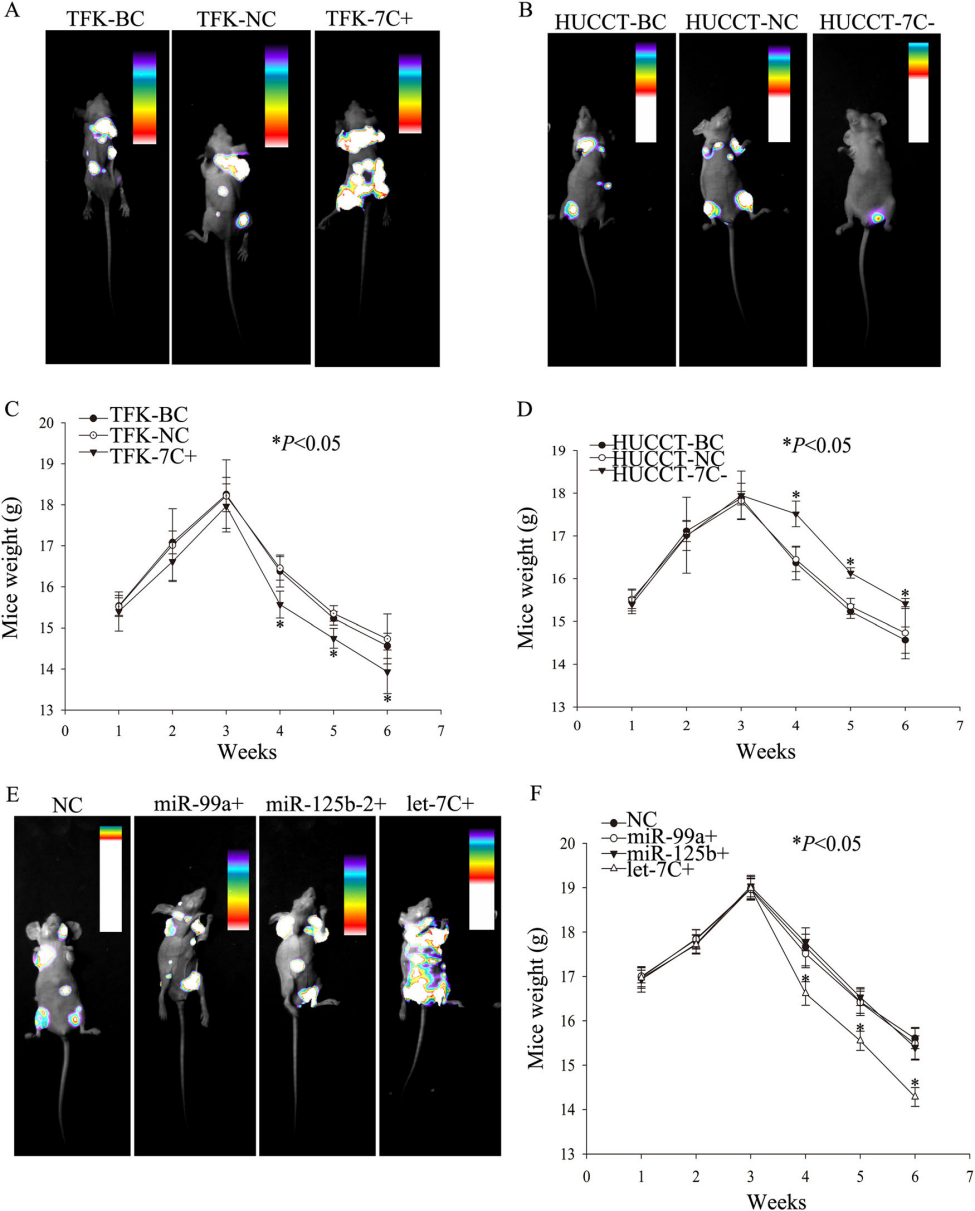
Aberrant expression of let-7c enhances metastasis in vivo. Live imaging of a distant metastasis model of lentivirus-transduced TFK-1 cholangiocarcinoma cells (**a**) and HUCCT-1 cells (**b**). **c**, **d** Body weights per mouse of the distant metastasis model. **e** Live imaging of distant metastasis model of lentivirus-transduced TFK-1 cholangiocarcinoma cells. **f** Body weights per mouse of the distant metastasis model. *P < 0.05; BC blank control group; NC negative control group; 7C+ let-7c-upregulated group; 7c− let-7c-downregulated group; miR-99a+ miR-99a-upregulated group; miR-125b-2+ miR-125b-2-upregulated group

To determine whether let-7c affects metastasis ability of cholangiocarcinoma in vivo, we injected the stable TFK-1 cells with high let-7c expression (and respective parental control cells) and HUCCT-1 cells with low let-7c expression (and respective parental control cells) into the tail vein of BALB/c nude mice in order to establish a distant metastasis mouse model. The results demonstrated that overexpression of let-7c promoted distant metastasis of cholangiocarcinoma cells as indicated by more metastasized foci (Fig. 5a) and less metastasized foci in the let-7c downregulated group (Fig. 5b) when compared with the negative control (NC) group. Using in vivo bioluminescence imaging, we found tumor foci mainly in the spine, bones of extremities, and lungs. Additionally, a significant decrease in the weight of mice was observed from the fourth week on (Fig. 5c, d). To additionally determine whether the other two miRNAs, miR-99a and miR-125b-2, that occur in the cluster with let-7c contribute to our observations, we generated a distant metastasis mouse model with miR-99a overexpressed cells or miR-125b-2 or let-7c overexpressed cells. The results showed that, compared with negative control group, there was no significant difference in metastatic foci in the miR-99a or miR-125b-2 upregulated group; only the let-7c upregulated group showed more metastasis foci (Fig. 5e, f). According to these observations, let-7c plays distinct roles in regulating invasion and migration of cholangiocarcinoma, in vitro and in vivo.

### Let-7c directly targets EZH2 and indirectly affects β-catenin via DVL3

**Fig. 6:**
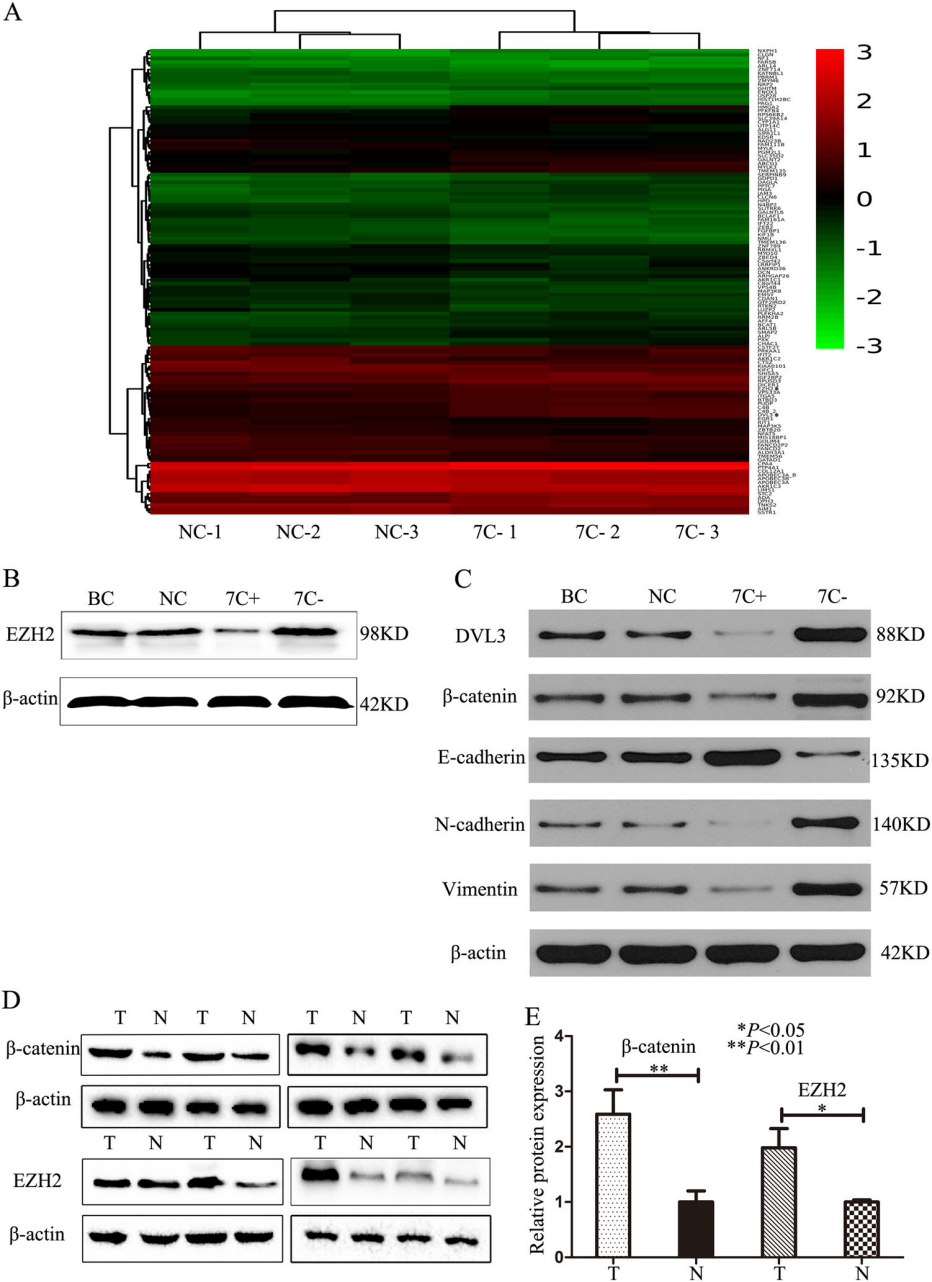
Let-7c directly targets EZH2 and indirectly affects β-catenin via DVL3 **a** The heat map of mRNA array showed the differential gene expression upon let-7c inhibition in HUCCT-1 cells. **b**, **c** The expression of DVL3, β-catenin, E-cadherin, N-cadherin, and Vimentin. β-actin was used as the loading control. All data were compared with the NC group. **d** The expression of β-catenin and EZH2 in paired cholangiocarcinoma and paratumor tissues from cholangiocarcinoma patients. **e** The quantification of western blot results. **P < 0.01; *N* Normal bile duct tissue; T cholangiocarcinoma (tumor group), BC blank control group; NC negative control group; 7C+ let-7c-upregulated group; 7c− let-7c-downregulated group

**Fig. 7:**
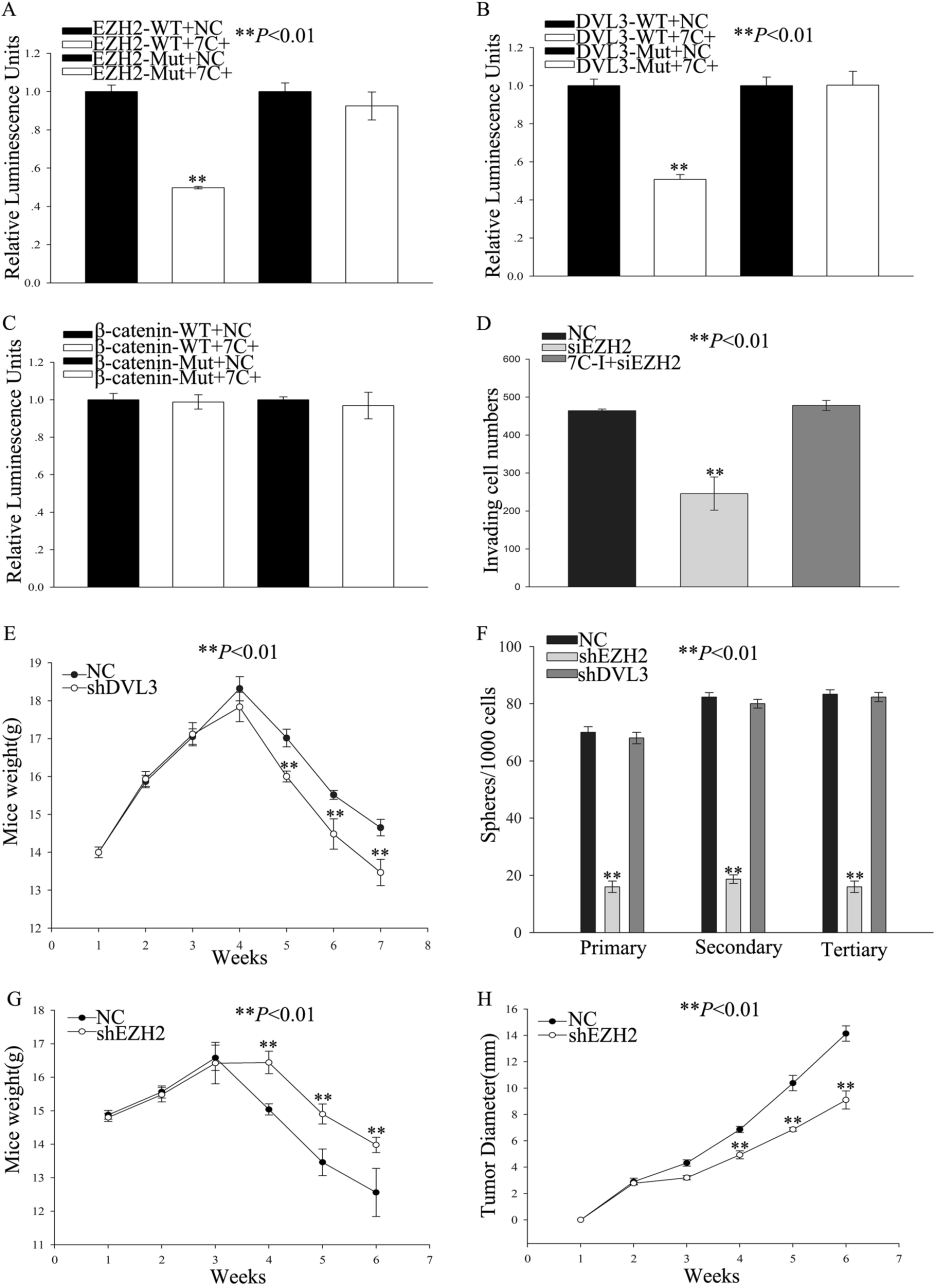
EZH2 and DVL3/β-catenin axis potentially participate in let-7c regulating the malignant biological behavior of cholangiocarcinoma. Luciferase reporter assay for (**a**) EZH2, (**b**) DVL3 and (**c**) β-catenin. **d** The effect of transfection with siEZH2 and let-7c downregulated plus siEZH2 on the invasive capacity of TFK-1 cells. **e** Weights per mouse of distant metastasis model. **f** The number of spheres per 1000 cells indicates the ability of shEZH2 suppressing capacity of sphere formation. **g** BALB/c nude mice weights and (**h**) tumor diameter of subcutaneous injected mice model. **P < 0.01. WT wild type; Mut mutant type; NC negative control group; 7C+ let-7c-upregulated group; 7C-I let-7c-downregulated group; siEZH2 Small Interfering EZH2; shEZH2 Small hairpin EZH2; shDVL3 Small hairpin DVL3

According to our results, let-7c affects migration, invasion, distant metastasis, tumor growth, and self-renewal capacities. Initially, based on TargetScan (TargetScan Human 7.0) analysis and published literature, we found that EZH2 is a direct target of let-7c in cancer cells^7,19^ and has a highly conserved let-7c binding site in its 3′-UTR (Supplementary Figure 2A). Also, EZH2 has been shown to be associated with promoting invasion and metastasis in different cancers^20–22^. *β-catenin*, an important gene in the Wnt/β-catenin pathway plays a role in epithelial-mesenchymal transition (EMT)^23^. It is regulated by Dsh (Dishevelled), which inhibits glycogen synthase kinase-3β for promoting the stabilization of β-catenin^24^. DVL3 contains one conserved let-7c binding site (TargetScan Human 7.0; Supplementary Figure 2B). In addition to Targetscan and published literature, we performed mRNA Arrays to detect the significantly deregulated and differentially expressed genes in let-7c downregulated in cholangiocarcinoma cells and in the negative control group. The result indicated that the mRNA expression of EZH2 and DVL3 was significantly increased in cholangiocarcinoma cells upon let-7c downregulation compared to the negative control group (*P* < 0.005 and 1.8125E−05, respectively) (Fig. 6a). In order to verify the result of mRNA array, we detected the expression of these two genes at the protein level, it demonstrated that overexpressed let-7c indeed resulted in a reduction in EZH2, DVL3 and β-catenin (Fig. 6b, c). In line with these results, let-7c inhibition led to an increase in the expression of EZH2 and DVL3 and β-catenin. To further validate whether EZH2 and β-catenin were post-transcriptionally regulated by let-7c, we detected the expression of EZH2 and β-catenin proteins in tissues from cholangiocarcinoma patients and respective adjacent non-tumor tissues via western blot and immunohistochemistry. The results from the paired samples showed that both, EZH2 and β-catenin, were overexpressed in cholangiocarcinoma tissues(Fig. 6d, e). Immunohistochemistry also showed that the protein expression levels of EZH2 (Supplementary Figure 2C and 2D) and β-catenin (Supplementary Figure 2E and 2F) were higher in tumor tissues than in adjacent non-tumor tissues. Moreover, we confirmed our results with a luciferase reporter assay. When the wild-type sequence of EZH2 was transfected into TFK-1 cells, we discovered that aberrant expression of let-7c lead to a reduction in luciferase activity. However, when TFK-1 cells were transfected with the luciferase reporter construct containing the mutated binding site in the 3’-UTR of EZH2, a decrease in luciferase activity was not observed (Fig. 7a). For DVL3, we got similar results (Fig. 7b). However, neither the wild-type sequence nor the mutated sequence of β-catenin showed a significant change in luciferase activity (Fig. 7c). These results demonstrate that *EZH2* and *DVL3* are direct target genes of let-7c, while *β-catenin* is not a direct target. However, let-7c may affect *β-catenin* via the upstream gene, *DVL3*.

### EZH2 and DVL3/β-catenin axis potentially participate in let-7c regulating the malignant biological behavior of cholangiocarcinoma

Previous studies have demonstrated that the let-7c—EZH2 axis is associated with migration and invasion abilities in cancer cells^7,19^. We next determined whether EZH2 and DVL3/β-catenin affect migration and invasion in cholangiocarcinoma. EZH2 and DVL3 siRNAs were transfected into TFK-1 cells to knock down expression followed by a transwell assay to determine the invasion ability. We found that knockdown of EZH2 expression decreased the numbers of invading TFK-1 cells compared with the negative control (Fig. 7d and Supplementary Figure 3A); furthermore, silencing of EZH2 in let-7c downregulated cells could recover the invasive capacity compared with negative control group (Fig. 7d and Supplementary Figure 3A). However, the invasive capacity of the siDVL3 group was not significantly different from the NC group (Supplementary Figure 3B and 3C). These results indicate that EZH2 knockdown can suppress the invasion of TFK-1 cells.

Besides, we transfected TFK-1 cells with lentivirus-based shDVL3/shEZH2 expressing vector and obtained a stable cell line with downregulated DVL3/EZH2 expression. We then injected these cells by tail vein injection into BALB/c nude mice to detect distant metastasis in vivo. Compared with the negative control group, we found that there were more distant metastasized foci only in the group with knockdown of DVL3 but not in the group with knockdown of EZH2 (Supplementary Figure 3D), in addition, the number of distant metastasized foci in the group with downregulated let-7c plus siDVL3 were as similar to the negative control group (Supplementary Figure 3E). Further, there was a significant weight reduction in mice from the DVL3 knockdown group compared with the negative control group from the fourth week on (Fig. 7e), but in let-7c downregulated plus siDVL3 group, there was no significant difference in weight reduction compared with negative control group (Supplementary Figure 3F). Taken together, our results reveal that the effect of let-7c on invasion and distant metastasis capacities of cholangiocarcinoma cells is, at least partially, mediated by EZH2 and the DVL3/β-catenin axis.

Moreover, we transfected HUCCT1 cells with let-7c mimics/inhibitor and detected the genes of mesenchymal to epithelial transition (MET). As shown in Fig. 6b overexpression of let-7c increased the expression level of E-cadherin and decreased the levels of N-cadherin and vimentin, the expression of DVL3 and β-catenin also decreased, when let-7c was upregulated. Furthermore, we also obtained the morphological changes in cholangiocarcinoma cells, which overexpressed let-7c (Supplementary Figure 3G). MET as a result of let-7c upregulation might thus inhibit the migration capacity of cholangiocarcinoma cells and thereby facilitate distant metastasis.

To confirm whether EZH2 affects tumorigenesis capacity of cholangiocarcinoma, we transduced a lentivirus shRNA vector into TFK-1 cells in order to silence *EZH2* and *DVL3* genes and repeated the sphere formation and tumor-initiating assays. We found that only the EZH2 shRNA cells formed smaller spheres compared with the NC cells (Supplementary Figure 4B and 4C). Furthermore, the number (Fig. 7f) of spheres increased faster in NC group than in the EZH2 shRNA group. Moreover, the cells with let-7c downregulation plus shEZH2 formed similar size spheres as the negative control group (Supplementary Figure 4D and 4E), over three passages, let-7c downregulation plus shEZH2 group formed a similar number of spheres compared to NC group. (Supplementary Figure 4F). However, the sphere formation ability of DVL3 shRNA group was not significantly different from the NC group. These observations were also applicable to the self-renewal capacity, wherein, the empty vector control group formed tumors much faster than the EZH2-shRNA group. The decrease in weights of BalB/c nude mice and tumor diameter were significantly different between the EZH2 knockdown group and NC group (Fig. 7g,h). Taken together, the results demonstrate that EZH2 and DVL3/β-catenin are involved in the malignant behavior of cholangiocarcinoma, are associated with migration, invasion, distant metastasis, sphere formation, and tumor initiation and are regulated by let-7c.

## Discussion

MiRNAs have been demonstrated in various cancer types to exert either tumor promoting or suppressive functions. The observed effects on potentially hundreds of genes can result in cell type and context dependent regulatory activities. The let-7c family of miRNAs was previously described as a tumor suppressor in various epithelial cancers such as gastric cancer^25^, colorectal cancer^26^ and hepatocellular carcinoma^13^. In our study, let-7c was expressed at low levels in advanced cholangiocarcinoma compared to adjacent non-tumor tissue. This result is in line with our data that conditionally let-7c overexpressing cholangiocarcinoma cell lines show reduced cell growth, mobility and wound healing compared to respective control cells. Both sets of data thus indicate a tumor-suppressive function of let-7c in local cholangiocarcinoma.

MiRNAs were not only recognized to regulate tumor growth but have also been recognized to play a role in other cancer-related processes such as metastasis^27–29^. The let-7 family members such as let-7b and let-7g have been shown to be involved in tumor metastasis^30,31^. Previously published data suggest that let-7c not only inhibits growth and invasion but also the metastatic capacity of various cancer types^11,32,33^. However, the link between let-7c and metastasis of cholangiocarcinoma is not clear. In our study, we demonstrated that overexpression of let-7c inhibits mobility, invasion and wound-healing capacities of cholangiocarcinoma cells in vitro, and similar results were reported by others^34^. In contrast to our cell culture experiments, however, tail vein injection of let-7c overexpressing cholangiocarcinoma cells led to rather higher tumor burden in distant extrahepatic sites compared to controls. To further elucidate the mechanisms of let-7c mediated pro-invasive capacity of cholangiocarcinoma cells in vivo, we analyzed predicted putative targets of the microRNA. We could demonstrate that let-7c directly targets the genes *EZH2* and *DVL3*. ShRNA mediated knockdown of DVL3 or EZH2 showed that only knockdown of expression of DVL3 promoted the pro-invasive activity of in vivo tail vein injected cholangiocarcinoma cells similar to let-7c overexpression. In the EZH2 knockdown group, we did not observe the promotion of pro-invasive activity. The dual role of pro-invasion and tumor suppression of let-7c in cholangiocarcinoma differs from results obtained in other cancers. A previous study demonstrated that reversion of EMT can promote proliferation and colonization of cancer cells in distant sites^35^. In our research, when upregulated let-7c, the expression of DVL3 and β-catenin were inhibited, in addition, the expression of N-cadherin and vimentin were also suppressed and the expression of E-cadherin was overexpressed. Moreover, we also observed the morphological changes in cholangiocarcinoma cells, which overexpressed let-7c. These data indicate that the cholangiocarcinoma cells have undergone reversion of EMT, and thus, facilitating the proliferation and colonization of cholangiocarcinoma cells in distant sites, in vivo. Further, the serum from patients with metastasis also showed increased let-7c compared to those with no metastasis. Nevertheless, considering the dual role of let-7c, caution should be exercised for application of let-7c as a therapeutic agent in cholangiocarcinoma.

In summary, our results reveal complex roles of the microRNA let-7c in human cholangiocarcinoma. Overexpression of let-7c inhibits the invasion capacity in vitro but enhances distant metastasis capacity in vivo. Furthermore, let-7c inhibits tumorigenic capacities of cholangiocarcinoma cells, including sphere formation and tumor-initiating capacity. This dual role in regulating cholangiocarcinoma can be imitated by regulating EZH2 and DVL3 expression. Thus, our observations provide strong experimental evidence regarding the involvement of let-7c in distant metastasis capacity of cholangiocarcinoma suggesting the microRNA as a novel biomarker for the identification of patients with metastatic disease.

## Materials and methods

### Tissues and serum

Bile duct cancer tissues were acquired from 13 cholangiocarcinoma patients whose postoperative pathological diagnoses were confirmed between May 2013 and March 2014 at the Department of Biliary-Pancreatic Surgery, Affiliated Tongji Hospital (Hubei, China) (Supplementary table 1). The procedures to collect human samples were approved by the China Ethical Review committee.

### Cells lines

TFK-1 cell line were provided by Renyi Qin, the Affiliated Tongji hospital, China, and originally purchased from DSMZ, Braunschweig, Germany. The HUCCT-1 cell line was provided by Jianmin Wang, the Affiliated Tongji hospital. TFK-1 and HUCCT-1 cell lines were cultured in RPMI-1640 medium supplemented with 10% fetal bovine serum (FBS), l-glutamine, and 1% penicillin/streptomycin. The EGI-1 cell line was cultured in 90% MEM supplemented with 10% h.i. FBS, 2× MEM amino acids (both essential and non-essential), 4 mM l-glutamine and 1 mM sodium pyruvate. All cell lines were maintained at 37 °C in a 5% CO_2_ incubator.

### RNA extraction and RT-PCR

Total RNA was extracted from 13 cholangiocarcinoma tissues and their adjacent normal bile ducts using Trizol (Invitrogen). Total RNA from sera of patients and controls were extracted using the mirVANATM PARISTM Kit (Ambion, USA) as per enclosed protocol. Real-time polymerase chain reaction was carried out with 50 or 500 ng of total RNA using PrimeScript Reverse Transcription Kit (TAKARA, Dalian, China) for miRNA and mRNA analyses, respectively. Real-time fluorescence quantitative polymerase chain reaction was run with SYBR Premix ExTaq (TAKARA) for miRNA and mRNA analysis. RNU6B was used as the internal control for miRNA qPCRs. We used the CFX96 Real-time PCR system for amplification and detection. (Bio-Rad, Hercules, CA, USA).

### Transfections

TFK-1 or HUCCT-1 cells were seeded in six-well plates and transfected with let-7c mimic/inhibitor or scramble, negative control (NC) (RiboBio, China) at a final concentration of 50 nM using siPORT NeoFX Transfection Agent (Ambion). SiRNA and the NC were purchased from RiboBio (Guangzhou, China). The sequences of siRNAs are available in Supplementary table 2. For let-7c overexpression and knockdown, lentiviruses encoding sh-EZH2, sh-DVL3 and sh-β-catenin were purchased from Genechem (Shanghai, China). All transfections were carried out according to the manufacturers’ instructions.

### Wound healing assay

A total of 1 × 106 TFK-1 or HUCCT-1 cells were seeded per well, in six-well plates, and cultured overnight. The following day, cells were transfected with let-7c mimic, inhibitor or negative control. After 24 h of transfection, the cell layer was scratched with a sterile plastic tip, washed two times with PBS, and then cultured with medium containing 1% FBS for 24 h. The cell layer was photographed under a microscope.

### Invasion assay

We used transwell inserts (24-well inserts, 8 um pore size; Corning Inc. Corning, NY, USA) to detect the invasive capacity of cholangiocarcinoma cells in vitro. The inserts for the invasion assay were pre-coated with extracellular matrix gel (BD Biosciences, Bedford, MA, USA). The transfected TFK-1 or HUCCT-1 cells were cultured with serum-free medium overnight. The following day, transfected cells were re-suspended with medium containing 0.1% bovine serum albumin and transferred to the upper chambers of the transwells. The lower chambers were filled with medium containing 10% FBS. Then the cells were incubated at 37 °C for 48 h. Later, the cells traversed through the membrane and adhered on the lower surface. Then, 0.4% paraformaldehyde and 0.1% crystal violet were used to fix and stain the cells, respectively. Finally, the stained cells were counted under a light microscope. Experiments were performed in triplicate.

### Distant metastasis model of cholangiocarcinoma cells in BALB/c nude mice

The BALB/c nude mice used for establishing the metastasis model were 4–6 weeks old, female, and purchased from the Animal Laboratory Unit of Peking Union Medical College (Bejing, China). We fed and housed the mice according to the institutional guidelines for animal care. Lentivirus-transduced cells (1 × 10^6^) were suspended in serum-free medium and then injected into the tail vein of mice. The weight of the mice was measured once per week. The extrahepatic tumors were visualized with Lumazone FA 2048 (Photometrics, USA).

### Heterograft experiment

Lentivirus (1 × 10^5^) transduced TFK-1 or HUCCT-1 cells were subcutaneously injected into the right armpit of BALB/c nude mice. The weight of the mice and the diameters of tumors were measured once a week.

### Suspension sphere culture and differentiation

Lentivirus-transduced TFK-1 or HUCCT-1 cells (1000 cells/ml) were cultured in suspension with serum-free DMED-12 (Hyclone, Logan, UT, USA), supplemented with B27 (1:50; Invitrogen, Carlsbad, CA, USA), 20 ng/ml epidermal growth factor (PeproTech EC, London, UK), 100 ng/ml basic fibroblast growth factor (PeproTech EC), and 100 ng/ml leukemia inhibitory factor (Chemicon, Billerica, MA, USA) as described elsewhere.^36^

### Luciferase reporter assay

According to Targetscan, we selected the 3′-UTRs of putative *EZH2/DVL3/β-catenin* genes whose sequence contained the predicted let-7c binding sites. We mutated three nucleotides in each of the EZH2/DVL3/β-catenin 3′-UTRs complementary to the let-7c seed region. TFK-1 cells were cultured in 24-well plates and transfected with 400 ng of either wild-type and one mutant pMIR/EZH2 or one mutant Pmir/DVL3 or one mutant pMIR/β-catenin plasmids with firefly luciferase, mixed with 100 ng Prl-TK vector (Promega, Madison, WI, USA) carrying renilla luciferase. This was done with either the let-7c mimic or the scramble control at a concentration of 50 nM each. Lipofectamine 2000 (Invitrogen) was used to perform the transfection. After 48 h, the relative luciferase activity was determined using the dual luciferase reporter assay kit (Promega).

### Statistical analyses

Data are presented as the mean ± s.e. The two-tailed student’s *t*-test was used to determine significance. Statistical analyses were performed by SPPS Software (version 19.0). A *P*-value of 0.05 was considered as significant.

## Electronic supplementary material

Supplementary Figure 1

supplementary Figure 2

Supplementary Figure 3

Supplementary Figure 4

supplementary table 1

supplementary table 2

Supplementary Figure Legends
