# Supplementary figures and images for: Let-7c inhibits cholangiocarcinoma growth but promotes tumor cell invasion and growth at extrahepatic sites
Source: Cell Death Dis. 2018 Feb 14;9(2):249. doi: 10.1038/s41419-018-0286-6 (PMC5833708; doi:10.1038/s41419-018-0286-6)

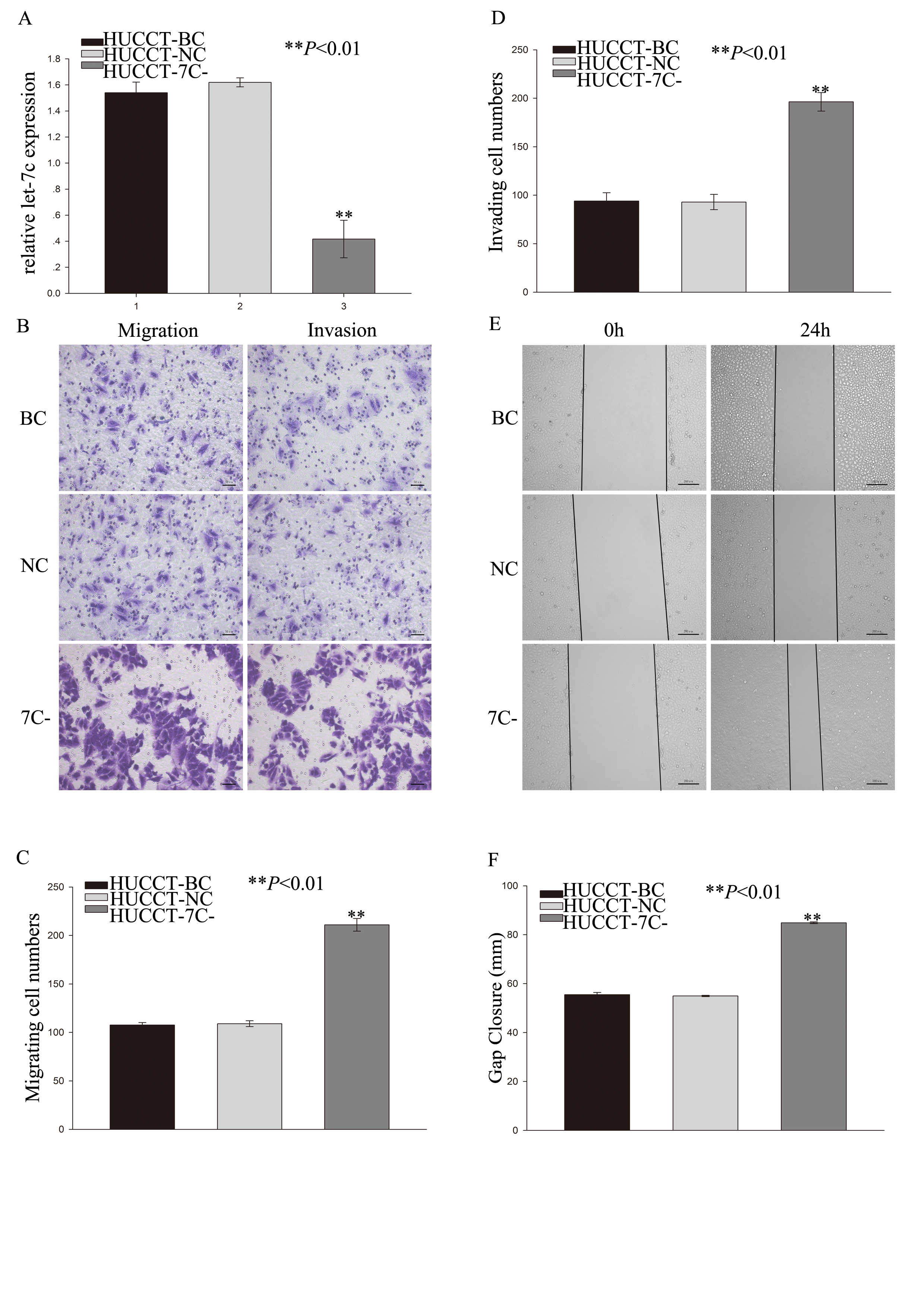

Supplement: Supplementary file 1 — Supplementary Figure 1 [file 41419_2018_286_MOESM1_ESM.tif]

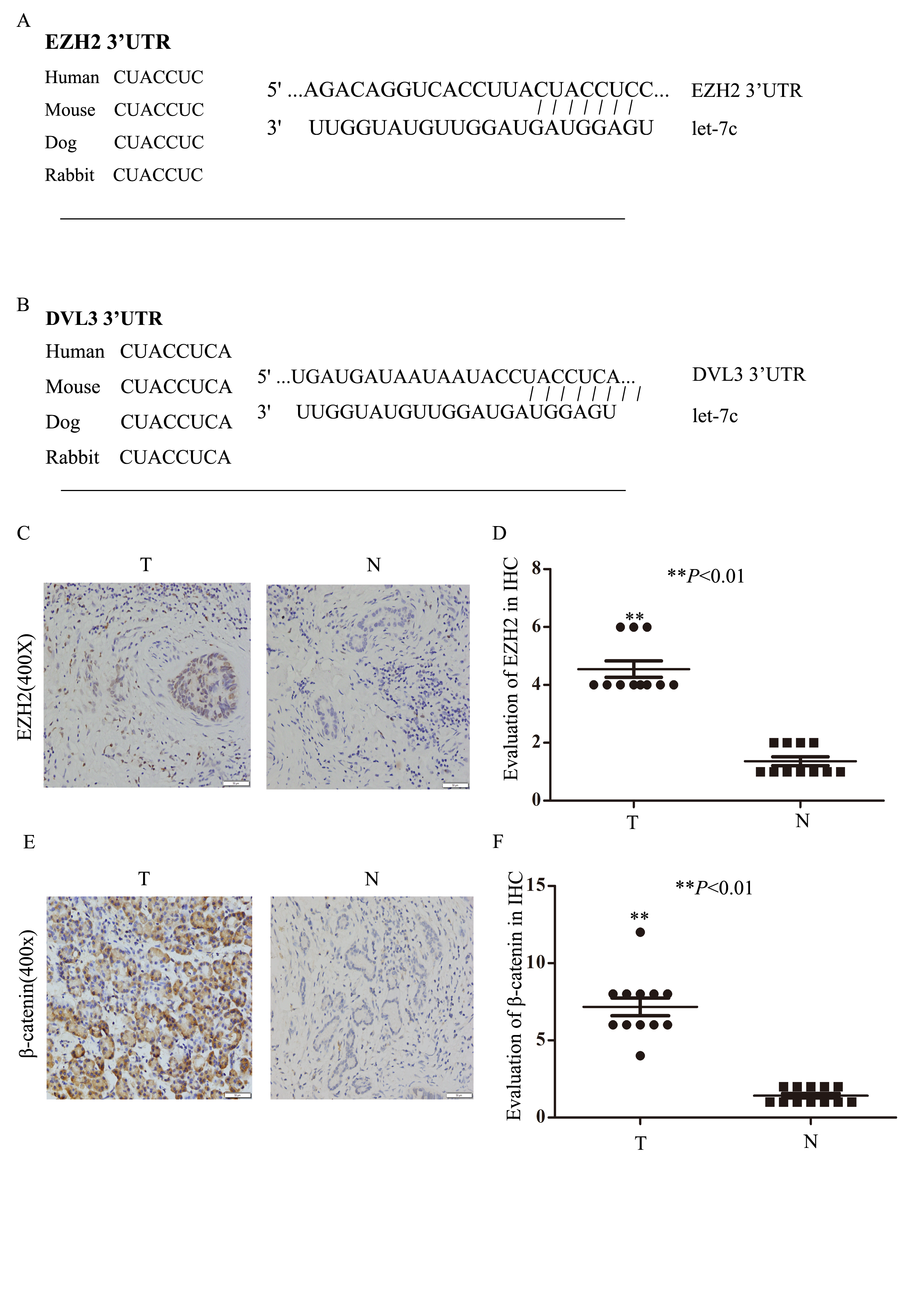

Supplement: Supplementary file 2 — supplementary Figure 2 [file 41419_2018_286_MOESM2_ESM.tif]

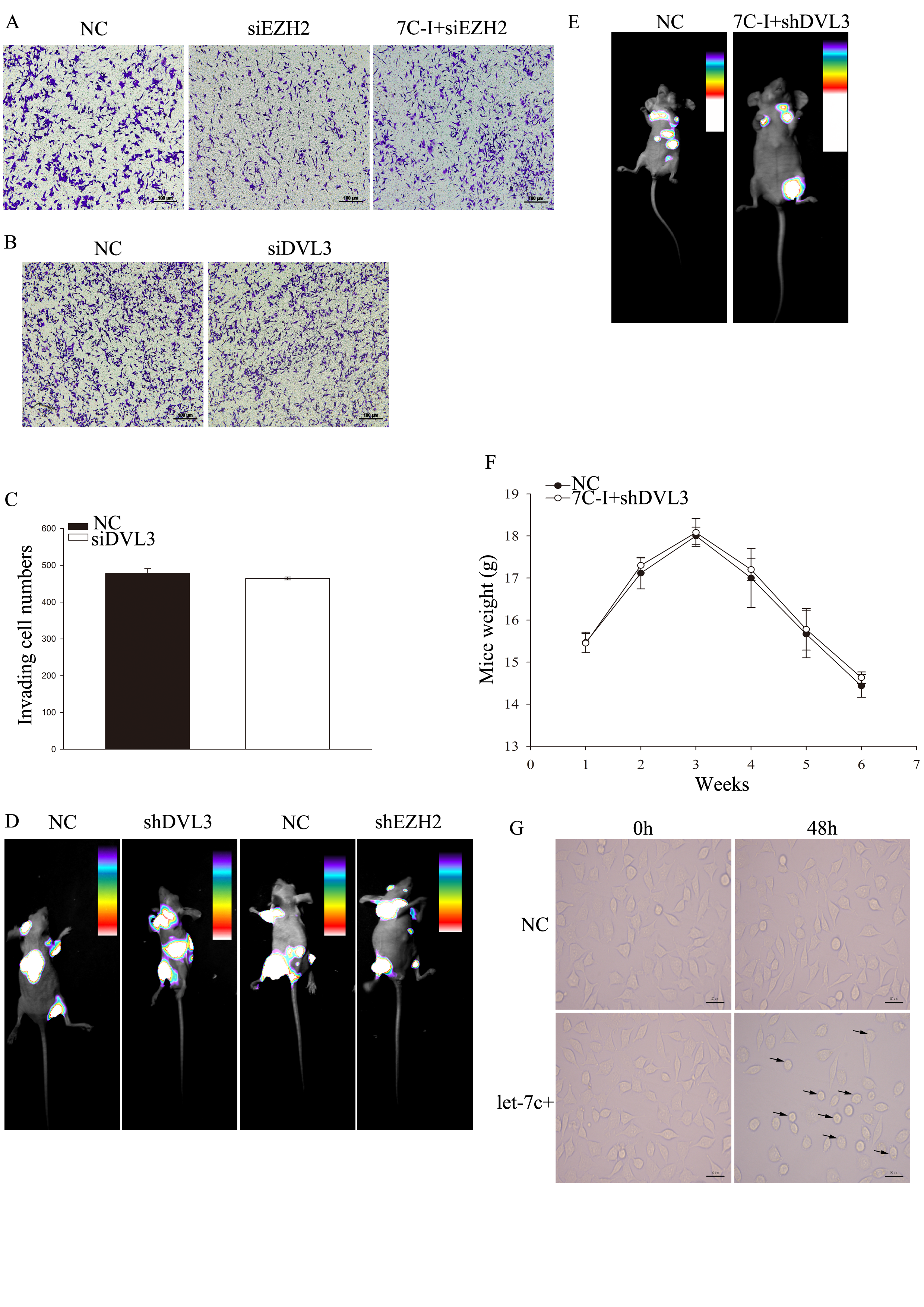

Supplement: Supplementary file 3 — Supplementary Figure 3 [file 41419_2018_286_MOESM3_ESM.tif]

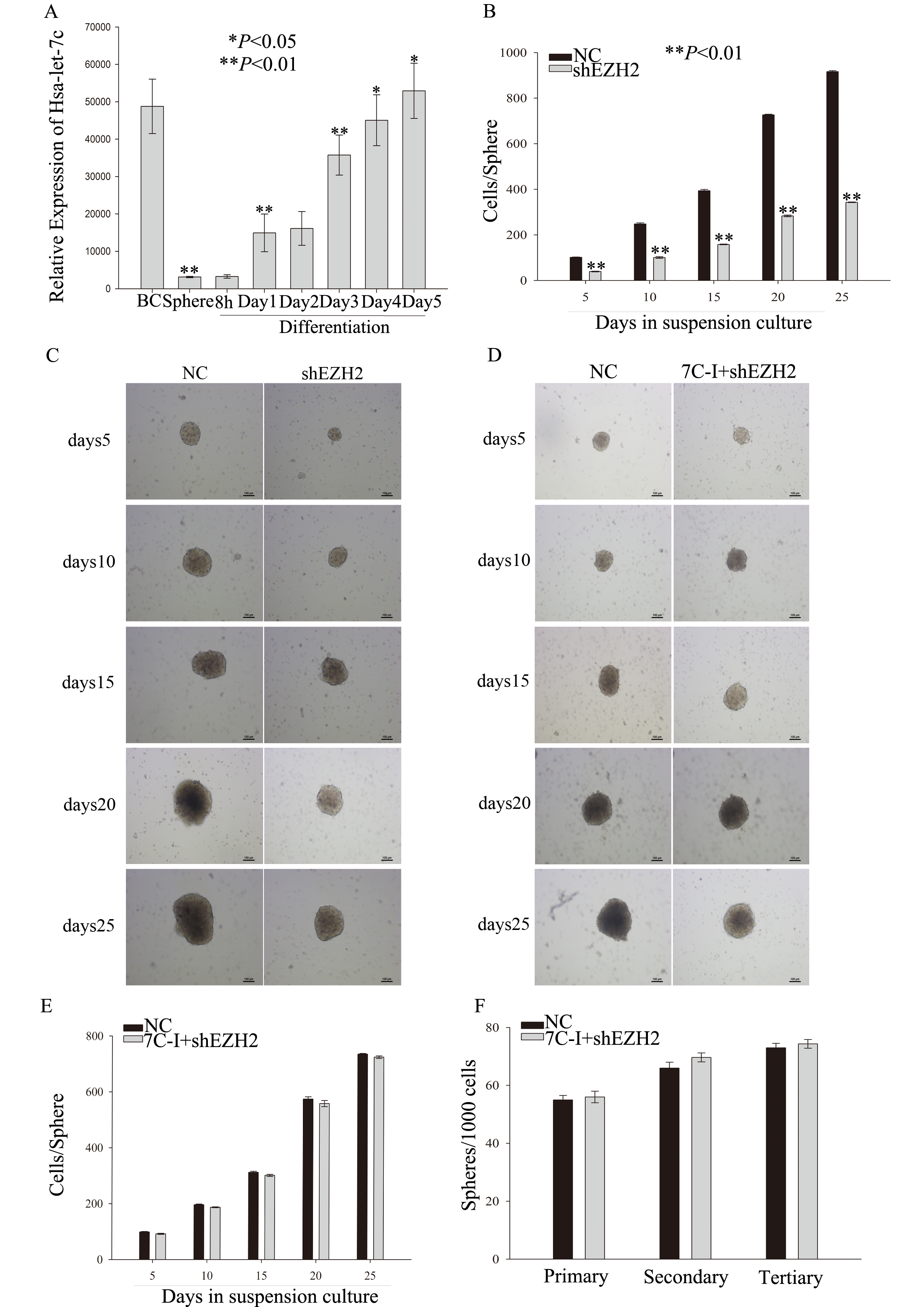

Supplement: Supplementary file 4 — Supplementary Figure 4 [file 41419_2018_286_MOESM4_ESM.tif]
