# Supplementary material for: Let-7c inhibits cholangiocarcinoma growth but promotes tumor cell invasion and growth at extrahepatic sites
Source: Cell Death Dis. 2018 Feb 14;9(2):249. doi: 10.1038/s41419-018-0286-6 (PMC5833708; doi:10.1038/s41419-018-0286-6)
Supplement: Supplementary file 5 — supplementary table 1 [file 41419_2018_286_MOESM5_ESM.doc]

**Supplemental Table 1 Correlation of let-7c expression in tissue with patients’ clinicopathological variables in 13 cases of cholangiocarcinoma**

|  |  | **Let-7c** | |
| --- | --- | --- | --- |
| **Variables** |  | **T** | **N** |
| Gender |  |  |  |
| Male | 9 | *P=*0.203 | *P=*0.485 |
| Female | 4 |  |  |
| Age(years) |  |  |  |
| <65 | 5 | *P=*0.289 | *P=*0.901 |
| ≥65 | 8 |  |  |
| Differentiation |  |  |  |
| Poor | 9 | *P=*0.648 | *P=*0.242 |
| Moderate+Well | 4 |  |  |
| Distant metastasis |  |  |  |
| Yes | 6 | **P=*0.043 | *P=*0.179 |
| No | 7 |  |  |
