# Supplementary material for: Let-7c inhibits cholangiocarcinoma growth but promotes tumor cell invasion and growth at extrahepatic sites
Source: Cell Death Dis. 2018 Feb 14;9(2):249. doi: 10.1038/s41419-018-0286-6 (PMC5833708; doi:10.1038/s41419-018-0286-6)
Supplement: Supplementary file 6 — supplementary table 2 [file 41419_2018_286_MOESM6_ESM.docx]

**Supplemental Table 2.** Primers and sequence used for Si-RNA transfection, and antibody used for western blot and immunohistochemistry

| **Gene** | **Forward** | **Reverse** |
| --- | --- | --- |
| Si-RNA |  |  |
| EZH2#1 | GAAUGGAAACAGCGAAGGAdTdT | dTdTCUUACCUUUGUCGCUUCCU |
| #2 | CCAUGUUUACAACUAUCAAdTdT | dTdTGGUACAAAUGUUGAUAGUU |
| #3 | GCUGAAGCCUCAAUGUUUAdTdT | dTdTCGACUUCGGAGUUACAAAU' |
| DVL3#1 | GGAUGACAAUGCCAAGCUAdTdT | dTdTCCUACUGUUACGGUUCGAU |
| #2 | CCACGUGGUUGCUUCACAUdTdT | dTdTGGUGCACCAACGAAGUGUA |
| #3 | CGACCCAGCUAUAAGUUCUdTdT | dTdTGCUGGGUCGAUAUUCAAGA |
| β-catenin#1 | GGUGGUGGUUAAUAAGGCUdTdT | dTdTCCACCACCAAUUAUUCCGA |
| #2 | GCCACAAGAUUACAAGAAAdTdT | dTdTCGGUGUUCUAAUGUUCUUU |
| #3 | GCUGAAACAUCAGUUGUAdTdT | dTdTCGACUUUGUAGUCAACAU |
| WesternBlot | | |
| EZH2 | | 1:250 |
| DVL3 | | 1:500 |
| β-catenin | | 1:250 |
| E-cadherin | | 1:500 |
| N-cadherin | | 1:250 |
| Vimentin | | 1:500 |
| β-actin | | 1:1000 |
| Immunohistochemistry | | |
| EZH2 | | 1:100 |
| β-catenin | | 1:100 |
