# Supplementary material for: Let-7c inhibits cholangiocarcinoma growth but promotes tumor cell invasion and growth at extrahepatic sites
Source: Cell Death Dis. 2018 Feb 14;9(2):249. doi: 10.1038/s41419-018-0286-6 (PMC5833708; doi:10.1038/s41419-018-0286-6)
Supplement: Supplementary file 7 — Supplementary Figure Legends [file 41419_2018_286_MOESM7_ESM.doc]

**Supplementary figure 1. Aberrant expression of let-7c inhibits migration and invasion of cholangiocarcinoma cells *in vitro***

(**A**) Validation of let-7c expression in HUCCT-1 cells following treatment with inhibitor. (**B**) Downregulated let-7c expression facilitated invasive capacity of HUCCT-1 cells. Increased migration and invasion in HUCCT-1 cells with downregulated let-7c expression. Numbers of (**C**) migrating and (**D**) invading cells. (**E**) Wound-healing assay with transfected HUCCT-1 cells. (**F**) The extent of gap closure in wound-healing assay showing.**P<0.01 Abbreviation: BC=blank control group; NC=negative control group; 7c-=let-7c-inhibitor group.

**Supplementary figure 2 Let-7c directly targets EZH2 and indirectly affects** **β-catenin via DVL3.**

Predicted let-7c binding sites in 3’UTRs of EZH2 (**A**) and DVL3 (**B**) with sequence complementarity and phylogenic conservation of 8 nt seed sequence. Immunohistochemistry to evaluate expression of EZH2(**C** and **D**) and β-catenin(**E** and **F**)in cholangiocarcinoma tissues and normal bile duct tissues. **P<0.01 Abbreviation: N=Normal bile duct tissue; T=cholangiocarcinoma (tumour group).

**Supplementary figure 3. EZH2 and DVL3/β-catenin axis potentially participate in let-7c regulating the malignant biological behavior of cholangiocarcinoma**

(**A**) The effect of transfection with siEZH2 and let-7c downregulated plus siEZH2 on the invasive capacity of TFK-1 cells. (**B**)The effect of transfection with siDVL3 on the invasive capacity of TFK-1 cells. (**C**) Numbers of invading cells. (**D**) Live imaging of a distant metastasis model of shEZH2 and shDVL3 transduced TFK-1 cells. (**E**) Live imaging of distant metastasis model of let-7c downregulated plus shDVL3 transduced cholangiocarcinoma cells. (**F**) Mice weight of distant metastasis model. (**G**) Cell morphology changes of let-7c overexpression. Abbreviations: NC=negative control group; 7c-I=let-7c-down-regulated group; siEZH2=Small Interfering EZH2 group; siDVL3=Small Interfering DVL3 group; shEZH2= Small hairpin EZH2 group; shDVL3= Small hairpin DVL3 group.

**Supplementary figure 4 EZH2 and DVL3/β-catenin axis potentially participate in let-7c regulating the malignant biological behavior of cholangiocarcinoma**

(**A**) Expression of let-7c during sphere formation and differentiation culture. (**B**) Quantification of cell numbers per sphere. Sphere formation with shEZH2 (**C**) and let-7c downregulated plus shEZH2 (**D**) transduced TFK-1 cells at multiple time points. (**E**) Quantification of cell numbers per sphere. (**F**) The number of spheres per 1000 cells indicates the capacity for sphere formation. The data were compared with the negative control group. *P<0.05, **P<0.01 Abbreviations: NC=negative control group; 7c-I=let-7c-down-regulated group; shEZH2= Small hairpin EZH2 group.
